# Grand challenges in pediatric stroke

**DOI:** 10.3389/fstro.2023.1204718

**Published:** 2023-05-19

**Authors:** Natalie Ullman, Daniel J. Licht

**Affiliations:** Department of Neurology, The Children's Hospital of Philadelphia, Philadelphia, PA, United States

**Keywords:** pediatric stroke, congenital heart disease, sickle cell anemia, childhood stroke, thrombectomy

## Introduction

Stroke is a broad term encompassing arterial ischemic stroke, cerebral venous thromboembolism and infarction, and non-traumatic brain hemorrhage. Pediatric stroke is further divided into perinatal stroke when occurring in the first 28 days of life, and childhood stroke for patients 29 days to 18 years. While there are challenges across all subtypes of pediatric stroke, this discussion will focus on arterial ischemic stroke (AIS).

Pediatric AIS is less common than stroke in adults, occurring at a rate of 1.6–4.4 per 100,000 per year in children (Agrawal et al., 2009; deVeber et al., 2017; Lehman et al., 2018; Ferriero et al., 2019; Mallick et al., n.d.). This number is higher among newborns with a rate of about 10–37 per 100,000 per year (Agrawal et al., 2009; deVeber et al., 2017; Dunbar et al., 2020). Despite its relative infrequency, pediatric stroke is a significant cause of lifelong morbidity (Greenham et al., 2016). Outcomes vary on infarct location and size, but up to 80% of children who have suffered a stroke develop hemiparesis or other motor impairments (deVeber et al., 2000; Ganesan et al., 2000). Many have cognitive deficits including lower intelligence quotient scores, issues with attention, processing speed, memory, and executive function (Westmacott et al., 2010; Hajek et al., 2014; O'Keeffe et al., 2014; Studer et al., 2014). Epilepsy occurs in about 15% of children who have suffered a stroke (deVeber et al., 2000). All of these sequela result in lower quality of life and lower rates of financial independence and independent living among childhood stroke survivors (Smith et al., 2015) many who have a normal life expectancy, so will live with these deficits for an average of 70 years. The most common risk factors for childhood AIS (CAIS) are focal cerebral arteriopathy (50%), cardiac disease (30%), arterial dissection (25%), and prothrombotic states (13%) (Mackay et al., 2011). The leading hypothesis on perinatal AIS is that it is the result of placental thromboembolism, though other theories exist (Bernson-Leung et al., 2018; Dunbar and Kirton, 2019).

## Part 1: acute arterial ischemic stroke diagnosis and treatment

### Diagnosing stroke

Stroke is the most common cause of focal neurologic deficits in adults, resulting in the development of well-oiled pathways for emergent assessment and imaging. Stroke alerts and similar protocols have been implemented in most large pediatric centers (Bernard et al., 2014; Tabone et al., 2017). However, not all children who are eligible for hyperacute interventions receive them due to delays in diagnosis and in transfer to pediatric stroke centers. Interestingly, the majority of children present to care within 4.5 h of symptom onset (Hutchinson et al., 2021), indicating a need for improvement among healthcare providers in early recognition of stroke symptoms and acquisition of rapid neuroimaging (**Grand Challenge 1**). The current delays are likely due in part to the relative infrequency of stroke in children, and higher frequency of stroke mimics such as migraine, seizure, functional neurologic disorder, and demyelinating diseases (Shellhaas et al., 2006; Hutchinson et al., 2021). However, the availability of interventions that could prevent death or a lifetime of disability necessitates that stroke be ruled out in any child presenting with focal neurologic deficits.

### Acute interventions

The Thrombolysis in Pediatric Stroke (TIPS) study was designed to determine safety, dose, and feasibility of intravenous tPA in children, but was closed for lack of accrual (Rivkin et al., 2015). Despite this, it resulted in the development of a network of pediatric stroke centers, through which the use of tPA in children was retrospectively assessed, and found to be safe but with questionable efficacy (Amlie-Lefond et al., 2020). While there are no pediatric trials demonstrating efficacy, tPA is considered in children meeting the adult criteria and present within 4.5 h, using the adult dose of 0.9 mg/kg, with the first 10% given as a bolus (Rivkin et al., 2016). Despite its widespread use in pediatric patients, there have been no studies evaluating optimal tPA dose in children, and some data suggest that higher doses may be needed due to developmental differences in plasminogen levels (Parmar et al., 2006). Tenecteplase (TNK) is rapidly replacing tPA in most adult centers due to numerous studies showing non-inferiority to tPA, a similar safety profile, and increased ease of administration (Kobeissi et al., 2023; Wang et al., 2023). Some studies even suggest higher reperfusion rates with TNK (Singh et al., 2023). However, there are no studies assessing use of TNK in children with AIS. In fact, there is no FDA-approved use for TNK in pediatrics, meaning pediatric hospitals would have to stock it specifically for stroke, which is an infrequent occurrence.

Thrombectomy has also proven to be safe and effective through numerous large randomized controlled trials (RCT) in adults within 6–12 h from time of last known normal (Fransen et al., 2014; Campbell et al., 2015; Goyal et al., 2015; Jovin et al., 2015; Saver et al., 2015; Bracard et al., 2016). The DEFUSE 3 and DAWN studies further expanded this window to 16 and 24 h, respectively, when selecting patients with favorable penumbra size using rapid perfusion imaging (Albers et al., 2018; Nogueira et al., 2018). Thrombectomy is considered standard of care for adults with large vessel occlusion, presenting within 24 h from last known normal, and with imaging showing salvageable brain tissue (Powers et al., 2019). Like adults, the natural history of children with large vessel occlusion is poor, with most experiencing lifelong disability or death (Bhatia et al., 2022), but comparable RCT data for thrombectomy in pediatrics does not exist. Case reports and a few larger cohort studies have demonstrated thrombectomy in children to be feasible and safe, with good neurologic outcomes in properly-selected patients (Tabone et al., 2017; Bigi et al., 2018; Bhatia et al., 2019; Sporns et al., 2020). The largest report by Bhatia et al., in 2019 was a systematic review of the literature from 1999 to 2019 and meta-analysis which included 113 mechanical thrombectomies in 110 children. They found 90.6% had good long-term neurologic outcomes. Death occurred in 2 patients and symptomatic hemorrhage in 1 patient. Importantly, the authors raise concern for publication bias and emphasize the need for prospective registries in pediatrics (Bhatia et al., 2019). The Save ChildS study was a multi-center retrospective cohort study that included 73 patients who underwent thrombectomy. They also found that the majority of patients had good neurologic outcomes, and there were similar rates of symptomatic hemorrhage to those reported in adult studies (Sporns et al., 2020). Save ChildS Pro is an ongoing multicenter prospective registry for thrombectomy in pediatrics (Sporns et al., 2021). The current American Heart Association guidelines suggest thrombectomy be considered in patients with disabling neurologic deficits, confirmed LVO on imaging, and of larger size (although not specified) (Ferriero et al., 2019), but is not yet considered standard of care. Limitations for the use of thrombectomy include patient size (weight limiting the amount of contrast available for use) and vessel size (distal branch clots).

Although TIPS closed due to lack of enrollment (Rivkin et al., 2015), a RCT for an acute intervention in pediatrics may be possible in the future with improved diagnosis and timely transport to pediatric stroke centers. However, for tPA and thrombectomy, a RCT in pediatrics will never be possible as the stroke community has lost equipoise. Due to compelling data in adults and success in pediatric cases, not offering these therapies would be unethical. It remains unknown how much and what type of data is required in pediatric stroke to make an intervention standard of care in the absence of a large RCT. Incorporating TNK into pediatric stroke care is also going to be a challenge. Replacement of tPA with TNK in adult centers will necessitate its use in pediatric patients treated in those settings or in combined adult-pediatric institutions. Pediatric hospitals will have to decide whether or not to switch their protocols in the absence of data from large RCT in pediatrics, but also with lack of strong evidence supporting continual use of tPA. At this time, the safety and optimal dose of TKN in children is not known. There are many ongoing trials in adults examining use of TNK in extended time windows, for minor stroke, and with thrombectomy, which are all questions that will also need to be addressed in pediatric patients as well.

### Neuroimaging

It is not known if the well-established adult time windows for reperfusion interventions are applicable to children. Preliminary data in mice (Faber et al., 2011) and looking at collaterals in aneurysmal subarachnoid hemorrhage (Moftakhar et al., 2015) suggest that younger patients may have more robust collaterals. If true, one would expect slower infarct growth rate allowing for longer time to reperfusion in children compared to adults. However, there is limited data on collateral status in pediatric ischemic stroke patients. A secondary analysis of 33 patients from Save ChildS study found over half of their cohort (19 patients) to have poor collaterals. The patients with good collaterals had smaller final stroke burden and slower early infarct growth, but there was no difference in clinical outcomes between the groups (Lee et al., 2021). More data is needed to understand if differences in vascular health and collateral blood flow in pediatric patients may result in differences in time to stroke completion. Rapid perfusion imaging is not readily available at many pediatric centers, and the protocols used in adult stroke have not been validated in children (**Grand Challenge 2**). Further research is needed to determine best perfusion imaging methods, reperfusion time windows in children, if there are patient/age-specific differences, and if there are different ranges for certain patient populations such as children with congenital heart disease.

It is also important to point out that for many pediatric patients, acute interventions are not accessible due to numerous barriers. These include few pediatric stroke centers and long travel times to care, lack of access to rapid neuroimaging, and lack of interventional neuroradiologists. Another important challenge is expanding access to these life-saving interventions to all eligible children, both within the US and globally. Training adult stroke practitioners to provide services for pediatric stroke patients could greatly improve this problem.

## Part 2: special populations in pediatric stroke

### Congenital heart disease

Cardiac disease is a significant cause of AIS, identified as a risk factor in about 30% of CAIS, with the majority of those patients having congenital heart disease (Mackay et al., 2011; Dowling et al., 2013). Congenital heart disease (CHD) occurs in 4–10 per 1,000 live births (Go et al., 2014). Within the last 50 years, surgical advancements have made it possible for patients with congenital heart disease to survive into adulthood, and it is estimated that in North America, ~1 in 150 adults are expected to have some form of CHD (Warnes et al., 2008).

CHD is a heterogenous term that includes both cyanotic and acyanotic structural heart defects. The risk of stroke in CHD is due to many factors that vary across the lifespan such as cardiac anatomies that predispose to clot formation and those that allow for paradoxical emboli. Certain cardiac procedures increase the risk for stroke, as does the need for mechanical circulatory support devices (Sinclair et al., 2015). Some data suggest that children with cyanotic CHD and single ventricle physiology are at highest risk for stroke (Asakai et al., 2015; Sinclair et al., 2015). However, prior studies have been limited by small numbers of patients with different CHD diagnoses.

We know that this stroke risk continues through adulthood. One large Swedish study found that adults with CHD had a 6 times higher risk of AIS than controls, despite having fewer traditional stroke risk factors of hypertension, diabetes mellitus, and hypercholesterolemia (Giang et al., 2020). A large Danish cohort study found CHD patients to have increased stroke risk as well as increased post-stroke mortality compared to the general population (Pedersen et al., 2019). Preliminary studies suggests that AIS risk may be higher in adult CHD patients with heart failure, recent MI, and co-morbid diabetes mellitus (Lanz et al., 2015). Atrial fibrillation is also common in adult CHD patients (Abiodun et al., 2016). More research is needed to understand which patients are at greatest risk and how this risk changes over time to guide screening and stroke prevention interventions (**Grand Challenge 3**). Further, it must be recognized that life-saving surgeries for certain CHD have only been available for 30–40 years, so the numbers of adult survivors are few, but the numbers are growing, and will continue to grow.

The time of greatest stroke risk in children with CHD is after cardiac procedures (Asakai et al., 2015). Despite many children with CHD having strokes while hospitalized, delays in diagnosis are common. Cardiac patients are often on anticoagulation, especially post-operatively, precluding the use of thrombolysis, but many would be thrombectomy candidates if a stroke was identified within the time window. However, the use of prolonged sedation and pharmacologic paralysis can mask seizures and hemiparesis. Furthermore, patients are often unable to undergo MRI due to medical devices such as pacing wires, or inability to travel off the cardiac unit. While HUS and portable HCT can be obtained at the bedside, these can miss up to 80% of acute strokes (Sinclair et al., 2015). This necessitates development of more sensitive bedside neuroimaging studies. Neuromonitoring with continuous electroencephalography (cEEG), near infrared spectroscopy, and transcranial Doppler is used in many cardiac units to detect changes in cerebral blood flow and guide neuroimaging. However, more research is needed to better understand which patients should be monitored, for how long, and if this impacts neurologic outcomes (Sinclair et al., 2015).

There is also some evidence that patients with CHD are more likely to have coexisting thrombophilia (Sträter et al., 1999). The mechanism for this is not understood, and currently routine thrombophilia screening is part of routine care for patients with CHD. More research is needed to understand if this is another factor contributing to stroke risk in this population, and should be screened for and mitigated if identified.

Finally, there remains an unanswered question about the clinical significance of a patent foramen ovale (PFO). One large study found PFO to be significantly more prevalent among patients with cryptogenic AIS compared to patients with known stroke etiology and healthy controls (Shih et al., 2021). However, we do not know whether, if the PFO is left unclosed, these patients are at increased risk for subsequent AIS. It is also not known whether PFO closure increases risk for developing atrial fibrillation later in life, which is well-known to be associated with AIS.

### Sickle cell anemia

Patients with sickle cell anemia (SCA) are another population with increased stroke risk throughout the lifespan. SCA is most common in sub-Saharan Africa where it is estimated that 230,000 affected children are born each year, which accounts for about 80% of all SCA cases globally. In comparison, about 2,600 and 1,300 children with SCA are born per year in North America and Europe, respectively (Rees et al., 2010).

The incidence of first stroke (hemorrhagic or ischemic) in patients with SCA is 0.61–0.761 per 100 patient years (Powars et al., 1978; Ohene-Frempong et al., 1998). A large cohort study in the US demonstrated that about 25% of patients with SCA will have had a stroke by age 45 (Ohene-Frempong et al., 1998). Notably, the risk for different types of stroke changes over the lifespan, with the risk of ischemic stroke being greatest during childhood and older adulthood, and risk for hemorrhagic stroke greatest during the second decade of life (Powars et al., 1978; Ohene-Frempong et al., 1998; Njamnshi et al., 2006; Kirkham and Lagunju, 2021). Furthermore, it is estimated that approximately 50% of patients with SCA experience “silent” cerebral infarcts (SCI) (DeBaun et al., 2020; Houwing et al., 2020), which are ischemic lesions identified on MRI that were not associated with an acute neurologic deficit. One small study reported a prevalence of SCI in up to 80% of patients when using a 7T MRI (van der Land et al., 2015). Although these are considered “silent” or subclinical, many studies have shown that SCI burden correlates with cognitive deficits (Houwing et al., 2020) and, along with stroke, may significantly alter educational attainment, employment status, and quality of life (DeBaun et al., 2020).

Studies have also shown that cerebral blood flow (CBF) and oxygen extraction fraction (OEF) are elevated in both children and adults with SCA (Prohovnik et al., 1989; Jordan et al., 2016; Fields et al., 2022). This is thought to be compensatory for the reduced arterial oxygen content due to chronic anemia. However, one study found that patients with SCA have increased CBF even compared to patients with chronic anemia, suggesting there may be other pathophysiologic mechanisms impacting cerebral autoregulation and stroke risk in patients with SCA that are not yet understood (Fields et al., 2022). It has also been shown that regions in the brain with high SCI burden correlate with regional increases in OEF (Fields et al., 2015).

Chronic transfusions aimed to lower hemoglobin S fraction to under 30% decrease stroke risk in SCA by 92% in patients identified to be at increased stroke risk by elevated velocities on transcranial doppler (TCD) (Adams et al., 1998). A subsequent study, demonstrated that discontinuation of chronic transfusions raised the stroke risk back to pre-treatment levels (Adams and Brambilla, 2005). Chronic transfusion therapy has also been shown to prevent accumulation of new SCI in patients with prior silent infarcts (DeBaun et al., 2014), and to reduce elevated to CBF and OEF (Guilliams et al., 2018). While effective, chronic transfusion therapy is not available in low-resource settings and many parts of the world with high rates of SCA. The SPRING trial conducted in Nigeria showed that low-dose hydroxyurea also decreases stroke risk in patients with SCA (Abdullahi et al., 2022), although is less effective than chronic transfusions (Ware and Helms, 2012). Furthermore, hydroxyurea therapy requires routine TCD screening and a life-long medication, which is still challenging in low-resource settings. Although interventions to decrease stroke in SCA exist, they are much less or not at all available to patients in sub-Saharan Africa where SCA burden is highest. It has also been shown that even within the United States, there are significant inequities among patients with SCA that limit access to care (Lee et al., 2019).

Another option for managing stroke risk in SCA is to cure SCA altogether. Allogenic hematopoietic stem cell transplant and gene therapy have recently entered the scene as curative therapies (Chakravarthy and Friedman, 2022). However, these are not without significant risks. It also requires access to a center that offers these treatments, and the ability to attend frequent medical appointments and prolonged hospitalizations. It is likely to be many decades before these options are accessible to the majority of patients with SCA. Further research should focus on not only preventing AIS in SCA, but on neuroprotective strategies to prevent subclinical or silent infarcts, with a focus on therapies that are accessible to all patients with SCA (**Grand Challenge 4**).

## Part 3: rehabilitation

It is generally thought that rehabilitation plays an important role in achieving optimal post-stroke recovery. However, current guidelines only recommend that patients undergo rehabilitation, without specification on techniques or duration (Hart et al., 2022). This is due to a general dearth of evidence on optimal rehabilitation strategies in pediatrics (Malone and Felling, 2020; Hart et al., 2022).

Small studies have shown constraint-induced movement therapy (CMIT) to improve upper limb function after perinatal stroke (Taub et al., 2011), although it is unknown whether these effects are sustained long-term (Mirkowski et al., 2019). I-ACQUIRE is a large RCT currently enrolling patients and examining the use of moderate dose (3 h per day) or high dose (6 h per day) of CIMT to usual treatment and will. Of note, I-ACQUIRE is enrolling patients with perinatal stroke, and a comparable large RCT does not exist for childhood AIS.

Transcranial direct current stimulation (tDCS) is a non-invasive brain stimulation technique that alters cortical excitability and may improve neuroplasticity after stroke (Stagg and Nitsche, 2011; Fleming et al., 2018). Initial pediatric studies have suggested that tDCS may be effective in improving lower limb function after perinatal stroke (Fleming et al., 2018). These results have not been replicated for upper limb function in pediatric patients (Fleming et al., 2018; Mirkowski et al., 2019), but there have been some studies suggesting efficacy in adult patients (Chhatbar et al., 2016). Repetitive transcranial magnetic stimulation (rTMS) is another non-invasive technique that works by inhibiting regional brain activity and increasing contralateral cortical excitability. A small trial of 10 patients demonstrated rTMS to be safe and suggested improvement in hand function in perinatal stroke patients (Kirton et al., 2008). The PLASTIC CHAMPS was a blinded randomized trial in which 154 patients with perinatal stroke received daily rTMS, CIMT, both, or neither in additional to motor learning therapy. The addition of rTMS, CIMT, or both doubled the chances of clinically significant improvement (Kirton et al., 2016). Similarly, tDCS can also be used with other forms of therapy, including CMIT or robotic-assisted therapy (Raess et al., 2022). Data from these small studies suggest tCDS and rTMS are safe, feasible, and potentially helpful in recovery after perinatal stroke. However, no studies have tested these interventions in childhood AIS. It also remains unknown if the improvement is long-lasting, how to best use in conjunction with other therapies (Mirkowski et al., 2019), whether the same parameters used in adults are applicable to children, and if patient-specific parameters should be considered (Gillick et al., 2014). TCDS and rTMS may be useful for non-motor realms of stroke recovery too. Both have been shown to be potentially effective in treating aphasia in adults (Fridriksson et al., 2018; Low et al., 2022; Stockbridge et al., 2023), but has not yet been explored for non-motor uses in pediatrics.

Robotic-assisted therapy and brain computer interfaces (BCI) are also being used more and more in rehabilitation post-stroke. The use of an exoskeleton has been shown to improve arm and hand function post-stroke (Biffi et al., 2018; Butzer et al., 2019). Other studies have shown promise in use of robotics or video-game-guided therapy in motor recovery (Fasoli et al., 2008; Valdés et al., 2018). For patients with severe motor deficits, brain computer interface-based interventions have the potential to be life-changing. These devices translate intention-driven electrical brain activity to control external devices (Jadavji et al., 2022). Small studies have shown that children are able to use BCI devices to operate power mobility devices (Floreani et al., 2022). This not only provides much needed independence, but also affords more opportunities for other areas of recovery through improved social participation. Currently these devices are not universally available and few providers are trained on using them, but they represent promise for patients with severe neurologic injury after stroke.

In summary, there is a wide range of rehabilitation techniques, interventions, and devices that are potentially helpful for stroke recovery. Like other areas of pediatric stroke, much of this has been extrapolated from adult stroke data and optimal regimens in children are not well-established. More research is needed to understand the type, timing, and duration of rehabilitation post-stroke, as well as how to individualize these interventions to the heterogenous pediatric stroke population (**Grand Challenge 5**).

## Summary of grand challenges in pediatric stroke

- Improvement in healthcare provider recognition of stroke symptoms allowing for acquisition of rapid neuroimaging and increased eligibility for acute interventions.
- Validation of rapid perfusion imaging protocols in children or identification of alternative imaging techniques to identify patients who will benefit from reperfusion therapies.

- Establish an understanding of the unique stroke risk associated with each type of congenital heart disease and how this risk changes across the lifespan.
- Development of neuroprotective strategies that prevent AIS and silent infarction in patients with sickle cell anemia that are accessible to all patients with SCA, including those in Sub-Saharan Africa.
- Determination of optimal type, timing, and duration of rehabilitation post-stroke.

## Author contributions

NU and DL contributed to the conception of the work. NU drafted the manuscript. DL made critical revisions. All authors contributed to the article and approved the submitted version.
